# “Stammering of the Urinary Organs” (Paget)

**Published:** 1895-02

**Authors:** J. Henry C. Simes

**Affiliations:** Philadelphia


### "STAMMERING OF THE URINARY ORGANS" (PAGET).

BY J. HENRY C. SIMES, PHILADELPHIA.

[*From University Medical Magazine.*]

Pathological condition of the genito-urinary tract, which occasion any interference or difficulty in performing the act of micturition, are frequently met with during the daily course of the physician's duties, and their importance on this account, aside from their own significance, is fully recognized by all surgeons and physicians. On the other hand, those cases in which there is no evident pathological change to account for difficult or impossible micturition are equally important, and, while they are not so often encountered, they occasionally are seen, and therefore their consideration is not without interest. Although the cases in which no pathological condition is demonstrable are not of so serious a nature as those in which the lesion is evident, yet when the frequency and importance of emptying the bladder is remembered, any hindrance to the normal passing of urine becomes a grave matter for the consideration of the physician, and may be a serious, if not a fatal, one for the patient; since the physician is only too fully aware of his imperative duty to relieve such a condition, and the patient is only too conscious of the condition in which inability to urinate places him.

It has been my experience with the class of patients who are sufferers of difficult micturition, not depending upon any evident pathological cause, but the so-called functional causes, that, while their actual suffering is not so great as those in which there is a positive pathological cause, yet the mental impression which their condition occasions is truly deplorable. They seem to live in a constant state of dread of what is to happen; they have lost all ambition in the affairs of life; they pass their time in consulting different physicians as to the nature of their trouble, and it is impossible to convince them that their condition is more one of inconvenience than one of a serious nature; indeed, like any of those conditions, whether due to pathological causes or to functional disturbances, affecting the organs of generation, there arises a peculiar mental state that occasions great depression, and may possibly terminate in melancholia.

Among those cases in which no pathological lesion can be ascertained to be present to account for the difficulty in micturition, those which Paget has so aptly named "stammering of the urinary organs," are of peculiar interest, and his description of a case of this kind is as follows: "The patient can often pass his urine without any trouble, especially at customary times and places; and, when he does so, then the stream is full and strong, and he has nothing the matter with him. But, at other times, he suffers all the distress that he might have with a very bad urethral stricture. He cannot pass a drop of urine, or, after passing a few drops, there comes a painful check, and the more he strains, the less he passes; and then complete retention may ensue, and overfilling of the bladder." While cases of this class are occasionally met with, they are not of very frequent occurrence, and therefore the two following may not be without interest:

H. L., aged 52 years, engaged in the manufacturing business. He gives the following history: Had always enjoyed fair health, although of not a very robust nature. Ten years ago, after having undergone a very severe mental strain, he first had some difficulty in passing his urine. At this time, he was examined for stricture, with negative result. Following the examination, he states, there was an attack of cystitis, and for the next three or four years he suffered with bladder troubles, and difficulty in passing his urine; indeed, until the present time he has been more or less troubled with interference in urinating.

Mr. L. first came under my observation three years ago, when he gave me the above history, and, upon further questioning, I ascertained that he was then suffering with some difficulty in passing his urine, the nature of which was that he was not able always to micturate; there was no pain along the genito-urinary tract, only the uncomfortable sensation of wishing to pass urine, and not having the power always to do it; there had never been complete retention of urine for any long period of time—that is, for twenty-four hours—but he had frequently passed ten or twelve hours without being able to empty his bladder, when the power would return, and he was able to urinate without any difficulty, passing his water in a full stream, and using no more than the ordinary muscular exertion. He cannot give any reason why he is at one time able to urinate in a perfectly normal manner, and at another time is powerless to pass a drop of urine; neither time nor place seems to have any influence over the attacks, nor does the condition of his health appear to control them; in fact, to him, as far as he has observed, he has never been able to connect this inability to empty his bladder with any cause whatsoever; he cannot foretell when an attack is going to occur, nor when it will pass away. The difficulty of urinating may continue daily for several weeks, and even two or three months, and again there may be an interval of months when he is capable of urinating in a perfectly normal manner. Whether the bladder contains a large or small amount of urine, it apparently has no influence in causing an attack, since under either condition the difficulty is liable to occur; nor does diet seem to affect his condition; in fact, from very careful questioning, I was unable to assign his affection to any apparent cause—that is, as to what occasioned the attacks.

Examination of the urethra, by means of sounds, gives no information as to the cause of his trouble; the canal is not oversensitive to the passage of the instrument; at the prostatic region of the urethra, the normal hyperesthesia is evident when the souud passes over the part; the bladder is not more than ordinarily sensitive to the presence of the instrument; the calibre of the urethra is fully up to the normal size, permitting a full-size sound to pass into the bladder without any hindrance; there is no spasmodic contraction of the muscles of the urethra occasioned by the introduction of the sound; indeed, the urethra gives no indication of any pathological condition, and the same may be said of the bladder. The prostate was examined, and appeared to be in a normal state, no enlargement or tenderness is evident to the finger when passed into the rectum. An examination of the urine gives no indication of any pathological change in any of the organs connected with the urinary system; its color, quantity and specific gravity are normal; there is no reaction to the sugar tests; albumin is not present; the urates are not in excess; the phosphates are slightly above the normal quantity, and there are a few more than the normal number of leucocytes present; also, the deposit of mucus is rather more than is observed in normal urine; no casts of any description are seen.

The above history and examination can but lead to one conclusion, and that is the condition of the patient is one which does not depend upon any evident pathological lesion of the genito-urinary organs, but is the result of some peculiar condition of the nervous system that affects the muscular system, which has for its function the emptying of the urinary bladder. As Paget writes, the organ stammers, and "stammering," he says, "in whatever organs, appears due to a want of concord between certain muscles that must contract for the expulsion of something, and others that must at the same time relax to permit the thing to be expelled." That the symptoms complained of by Mr. L. are due solely to this want of concord in the action of his muscular system connected with the act of micturition, is the only conclusion to be reached, and, therefore, the diagnosis of stammering of the urinary organs may very properly be applied to his case.

The second case is a younger brother of the above; he gives the following history: Several years ago, he first noticed some difficulty in urinating—that is, he was at times unable to pass his urine—and this difficulty occurred without any known cause; the attacks occurred at irregular intervals, between which he passed his urine in a normal manner. I may say that he gives a history very similar to that which I have above related in the case of his brother; but, unfortunately for him, shortly after he was first attacked with the symptom of interference in micturating, he had a fall while riding his bicycle, which occasioned him some pain in the perineum and penis; a surgeon was consulted, and the diagnosis of a stricture of large calibre of the urethra was made; treatment was begun for this lesion, and an attack of acute cystitis followed, terminating in chronic cystitis, from which he has never recovered. My connection with this case began three years ago, at the same time I had his brother under my care, and, upon examination, I found the case to be one of acute cystitis, occurring in a bladder which for several years suffered with chronic inflammation, and that these attacks of acute inflammation had, at irregular intervals, affected the organ since he was first treated for a stricture of large calibre. This complication of cystitis has, in a measure, masked the original trouble—the stammering of the organ—and has also added very much to his suffering. That the stammering still exists, I am satisfied from my observation of the case during the past three years, and, although the cystitis has never been entirely recovered from, notwithstanding the most persistent treatment, yet at times he has been almost free from any of its symptoms for a considerable period, but during these intervals of abatement in the cystitic trouble there have been, now and then, attacks of difficulty in passing water, which in every way resemble those described in his brother's case.

Examination of the urethra and bladder give no indication of any pathological condition which would account for the interference in urinating; there was no obstruction to the passing of a large-sized sound, except an over-sensitiveness at the neck of the bladder, due, undoubtedly, to the long-continued inflammation of this organ; the prostate seemed normal. Examination of the urine gave no indication of any morbid change in the kidneys; there were present the usual constituents of chronic cystitic urine-pus-cells, epithelium, phosphates, albumen and mucus.

I may here state that in both the above cases there is no history of there ever having been any venereal disease, nor is there any evidence, after careful examination, that the patients have ever suffered from such affections. They are both unmarried, and sexual excess has never been indulged in; indeed, I have every reason to think that they have both lived very continent lives, not only in regard to the sexual passion, but temperate in all things. There is no history or indication of any injury or disease of the genito-urinary system ever having existed during the life of either patient, except their present complaint.

In conclusion, a word upon the treatment of these cases of stammering of the urinary organs; and I wish I was able to offer something more encouraging than my experience with the two above cases. Everything which in any way presented the faintest hope of relief has been given a trial, and I can here say that both patients were fully competent, and only too willing to carry out all instructions, but nothing has ever been of any permanent benefit. Now and then a change in the therapeutics or method of treatment has raised their hopes, but it has always proved a temporary condition; sooner or later, there was a return of the old trouble, and a corresponding depression in the mental state of the patient. It would be useless, as well as unprofitable, to enumerate all the different therapeutic remedies which have been administered, and all the methods which have been employed in the treatment of these cases; it is sufficient to say that nothing has been of permanent benefit, and the advice given by Paget, some twenty-five years ago, holds good to-day; my experience in the treatment of these cases is in full accord with him. He writes, as follows: "The treatment of stammering urinary organs has difficulties similar and equal to those of treating stammering speech. The patient must try to educate himself to a calm control of his muscular power; and on any occasion of failure must get what help he can from such mental tricks as I have referred to. He should evade all risks of difficulty, and all the conditions in which he has suffered his worst failures. He should do anything rather than fail to pass his urine. He should not always yield to the first impulse to pass it, but should try to regulate the actions of the bladder to certain fixed hours of the day. And especially he should learn to use a catheter, not only that he may thus relieve himself in case of absolute need, but that he may be free from the enervating dread of helpless retention. He should keep his whole economy, and chiefly the secretion of urine, in the healthiest state he can; for, like all other stammering, or even in a greater degree than any other, that of the urinary organs is influenced by the condition of the general health."

